# Gestion du surpeuplement au Service d’accueil des urgences (SAU) du Centre Hospitalo Universitaire Gabriel Touré, Bamako au Mali

**DOI:** 10.11604/pamj.2022.41.4.28544

**Published:** 2022-01-03

**Authors:** Almeimoune Abdoulhamidou, Diop Madane Thierno, Mangane Moustapha, Démbele Seidou Alaji, Coulibaly Mahamadoun, Sogoba Youssouf, Cisse Mahamadou, Harouna Sangare, Sidy Yattara, Marie Casimire Mindzie Mintsa, Kassogue André, Diallo Boubacar, Diango Djibo Mahamane

**Affiliations:** 1 Département d´Anesthésie Réanimation et de Médecine d´Urgence, CHU Gabriel Toure, Bamako, Mali,; 2 Université des Sciences, des Techniques et des Technologies de Bamako (USTTB), Faculté de Médecine et d´Odontostomatologie, Bamako, Mali,; 3 Service d´Anesthésie Réanimation, CHU IOTA, Bamako, Mali,; 4 Service d´Anesthésie Réanimation, CHU Luxembourg, Bamako, Mali,; 5 Service de Neurochirurgie, CHU Gabriel Touré, Bamako, Mali,; 6 service d´Accueil des Urgences, Hôpital du Mali, Bamako, Mali,; 7 Département d´Anesthésie Réanimation et de Médecine d´Urgence, CHU Point G, Bamako, Mali

**Keywords:** Urgences, parcours de soins, surpeuplement, durée séjours, triage, Emergencies, care paths, overcrowding, length of stay, triage

## Abstract

**Introduction:**

confronté à un challenge quotidien d´engorgement des urgences, nous avons mené cette étude afin de déterminer les causes de la surpopulation du service des urgences et évaluer le processus d´orientation d´aval post soins.

**Méthodes:**

il s´agit d´une étude prospective sur un an au service d´accueil des urgences du CHU Gabriel Touré. Ce travail incluait tous les patients admis dans ledit service appartenant aux classes 3, 4, 5 de la classification clinique des malades aux urgences (CCMU) et dont la durée de séjour au service est supérieure ou égale à 24h. N´ont pas été inclus les patients consultants aux urgences et appartenant aux classes 1, 2, de la classification CCMU; les patients n´ayant pas de dossier médical bien établi, les patients décédés avant soins.

**Résultats:**

nous avons enregistré 19 571 recours au service d´accueil des urgences dont 44 cas d´afflux massif drainant 570 patients. Taux d´occupation des lits était de 108,03% dans notre service au même moment la moyenne générale à l´échelle de l´hôpital affichait 56%. Selon la classification CCMU, 83,75% des patients étaient CCMU3. Les patients neurolésés avaient représenté 557 cas. Les pathologies traumatiques avaient représenté 56,7% des patients contre 49,2% de pathologies médicales rencontrées.

**Conclusion:**

la durée moyenne de séjour était 63,59 heures et une durée max de 45 jours. Plus d´un quart des motifs de retard de mutation intra hospitalière était dû à la nécessite une surveillance spécifique ou des soins particuliers non faisable en hospitalisation conventionnelle.

## Abstract (en)

**Introduction:**

Emergency Department (ED) overcrowding is a major healthcare issue. The purpose of this study is to determine the causes of emergency department overcrowding and to evaluate intra-hospital transfer after initial treatment.

**Methods:**

we conducted a 1-year prospective study at the Emergency Reception Service of the Gabriel Touré University Hospital. All patients admitted to the Emergency Reception Service and belonging to classes 3, 4, 5 of the Clinical Classification of Emergency patients (CCEP) and whose length of stay in the department was greater than or equal to 24 hours were included in the study. Patients consulting the Emergency Department and classified as CCMU classes 1, 2, patients without a well-established medical record and patients who died before treatment were excluded by the study.

**Results:**

we recorded 19.571 calls to the emergency service, including massive influxes (a remarkable 44 times, 570 patients). Bed occupancy rate was 108.03% in our department; at the same time, the average bed occupancy in the Hospital was 56%. According to the CCEP classification, 83.75% of patients were CCEP3. Patients with neurological disorders were 557. Patients with a trauma accounted for 56.7%, compared with 49.2% of medical disorders encountered.

**Conclusion:**

the average length of stay was 63.59 hours, with a maximum length of 45 days. More than a quarter of the reasons for delayed intra-hospital transfers were due to the need for specific surveillance or specific treatments that were not feasible in conventional hospitalization.

## Introduction

La prise en charge des urgences médicales est l´une des missions principales des établissements de santé. Ces derniers, au travers des services d´urgence tentent de répondre à une demande exponentielle de soins non programmés, ce qui pose un problème d´engorgement des structures d´urgence [1]. En outre, pendant la même période, le maintien de la polyvalence de la médecine d´urgence a fait apparaître des difficultés dans la gestion des flux accentués par l´augmentation de la demande de soins et la pénurie en personnels soignants [2,3]. L´accroissement des recours aux services des urgences constitue un problème constant dans les pays industrialisés comme dans les pays en voie de développement évoqué déjà par plusieurs auteurs [2-5].

Au Mali la pratique de la médecine d´urgence est caractérisée par l´absence de médecine préhospitalière. Le CHU Gabriel Touré, principal hôpital à vocation d´urgence de la capitale malienne dispose du seul service d´accueil des urgences (SAU) du pays. A ce titre, il draine toutes les urgences médico-chirurgicales de la ville de Bamako et fait face à une grande sollicitation de la part des hôpitaux de l´intérieur du pays et des pays voisins. Ce service est constamment surpeupler sous le flux constant des recours de soins et créant ainsi un engorgement. Pour continuer d´assurer sa mission d´accueil des urgences, il devient indispensable d´orienter les patients stabilisés vers d´autres services spécialisés pour la continuité de leur prise en charge afin de le désengorger. Confronter à ce challenge quotidien d´engorgement au service des urgences nous avons décidé de mener cette étude dont les objectifs étaient de déterminer les causes de la surpopulation du service des urgences connaitre le profil pathologique de nos patients et évaluer le processus d´orientation d´aval post soins.

## Méthodes

**Type d´étude**: nous avons mené une enquête observationnelle de type descriptive à collecte rétrospective allant du 1er octobre 2018 au 30 septembre 2019 soit une période de 12 mois.

**Figure 1:**
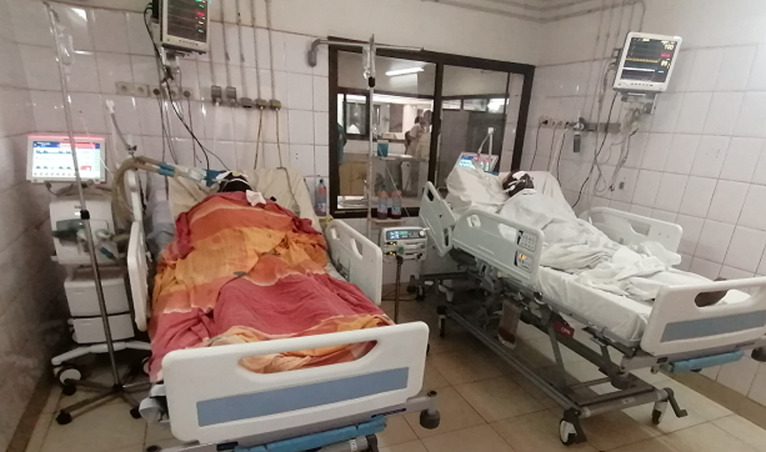
salle d'accueil des urgences vitales

**Cadre de l´étude:** cette enquête s´est déroulée au Service d´Accueil des Urgences (SAU) du CHU Gabriel Touré. Bien vrai que ce service ait une vocation de structure d’urgence traumatologique et mère-enfant, il reçoit prioritairement les patients adultes à l’exclusion des urgences médico-pédiatriques et obstétricales. Ces derniers sont reçus sur des aires d´accueil dédiées séparées du SAU. L´organisation structurelle du SAU comprend une zone de tri, une salle de déchoquage de deux lits (Figure 1), 02 unités d´hospitalisation de courte durée (UHCD) de 08 lits, 08 box de consultation, une zone d´attente assise extensible, 01 bloc opératoire d´urgence, 01 laboratoire délocalisé d´analyse sanguine, une salle de radiologie, Un secteur administratif (02 bureaux et un amphithéâtre). Durant la période de cette étude le bloc opératoire et le laboratoire étaient fermés pour travaux.

**Critère d´inclusion**: ce travail incluait tous les patient admis au SAU appartenant aux classes CCMU 3, 4, 5 [6] et dont la durée de séjour au service est supérieure ou égale à 24h.

**Critère de non inclusion**: n´ont pas été inclus les patients consultants aux urgences et appartenant aux classes 1, 2, de la classification CCMU, les patients décédés avant soins. Les données ont été recueillies à l'aide d'une fiche d'enquête individuelle préétablie à partir des dossiers médicaux et du registre d´admission.

**Variables étudiées**: âge, sexe, provenance, motif de recours et d´admission, statut CCMU, score de Glasgow. Les examens complémentaires biologique et radiologique réalisés, les diagnostics de sortie, le triage, la sectorisation des patients, gestion des mutations hospitaliers, les motifs de retard d´orientation des patients, durée du séjour dans le service.

**Analyse et saisie**: les données ont été saisies et analysées sur le logiciel SPSS version 20.0. Le traitement du texte, des tableaux et des graphiques a été réalisé grâce aux logiciels de la suite Office 2016 de Microsoft: Word et Excel. Les résultats ont été présentés sous forme de tableau pour présenter les fréquences, les moyennes et les écart-types des variables quantitatifs.

**Considération éthique**: afin de garantir la confidentialité des informations personnelles des patients, les données ont été recueillies sur des fiches d´enquête anonymisées tout au long de cette enquête.

## Résultats

**Figure 2:**
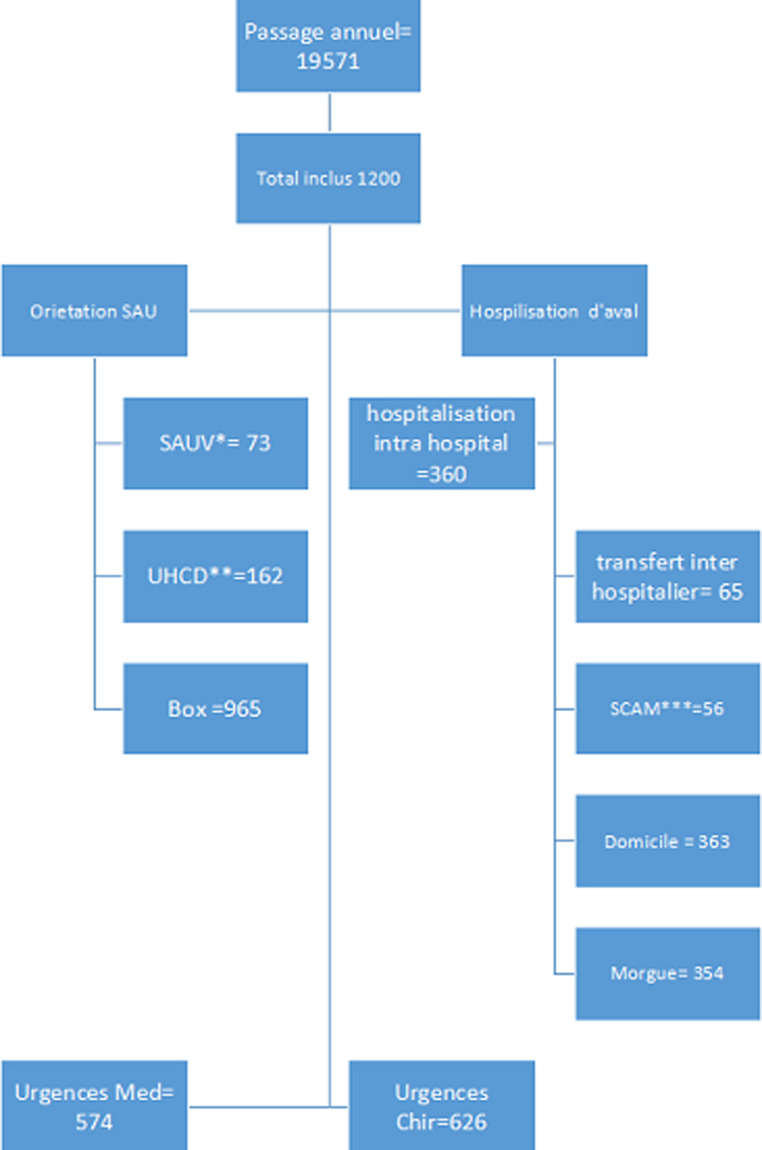
diagramme de flux des admissions

Durant notre période d´étude, nous avons enregistré 19 571 recours au service d´accueil des urgences du CHU Gabriel TOURE dont 44 cas afflux massif à type d´accidents catastrophiques à effet limité (ACEL) ayant drainé 570 victimes. Le taux d´occupation des lits était de 108,03% dans notre service au même moment la moyenne générale à l´échelle de l´hôpital affichait 56%. Ainsi 1200 patients ont été inclus dans notre travail reparti selon la Figure 2. Le sex ratio H/F était de 2,37. La moyenne d´âge était de 38,51 ans, avec des extrêmes de moins d´un an à plus de 100 ans. Les enfants avaient représenté 11,6% (n=140) des admissions, dans cette population la tranche d´âge 8 à 15 ans était la plus représentée soit 85% des cas. Vingt-quatre pourcent (24%) des sujets (n=297) avait plus de 60 ans. Les patients venaient majoritairement de leur domicile à 44% (n=528), d´une structure sanitaire dans 31,8% (n=382) et pour 24,2% ils ont été transportés par la protection civile. Quelques soit le moyen de transport, il n´y avait pas de médicalisation préhospitalière.

**Figure 3:**
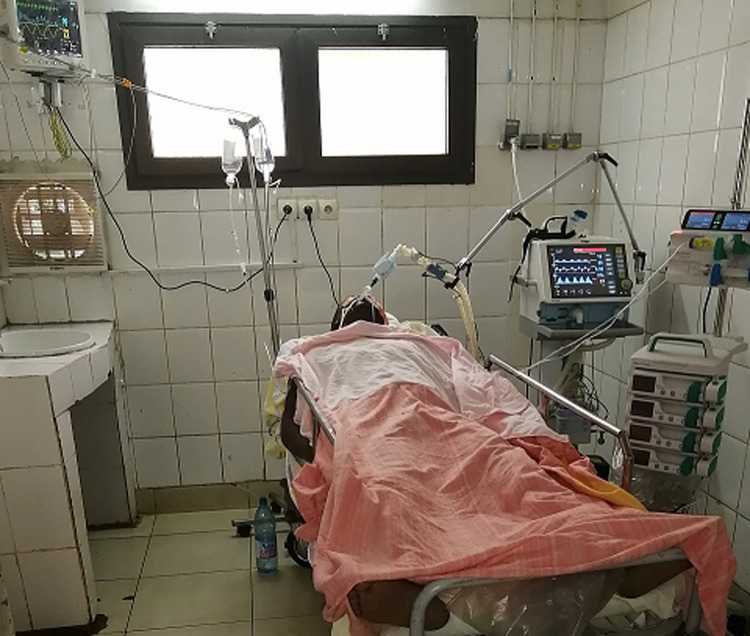
box de consultation converti en SAUV

**Figure 4:**
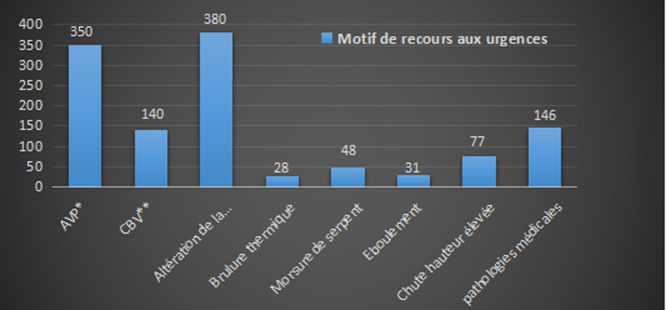
motif de recours aux urgences

Le triage et l´orientation ont été effectués sur la base de la classification CCMU. Ainsi 83,75% (n=1005) des patients inclus dans notre étude était CCMU3. Les classes CCMU4 et CCMU5 ont représenté respectivement 13,9% (n=167) et 2,5% (n=30). Selon le score de Glasgow, 70% (n=841) des patients était conscience à l´admission contre 30% en altération de la conscience dont 8,5% ( n=102) dans un coma profond. Ce taux élevé de patient comateux nous obligeait à convertir trois box de consultation en salle d´accueil des urgences vitales (SAUV) (Figure 3). Les motifs d´admission étaient divers et variés tels que illustrés par la Figure 4. Nous avons effectué 1 411 examens d´imagerie médicale. Elles avaient consisté à la réalisation de 842 examens tomodensitométriques (TDM) principalement chez les traumatisés crâniens en l´absence totale de moyen transport médical intra et inter hospitalier. La radiographie standard a été réalisé chez 479 malades en intra-muros. Dans 302 cas, il s´agissait d´une radiographie thoracique et pour 177 cas des radiographies des os long. Un bilan biologique a été réalisé 4 606 fois, à savoir l´hémogramme 942 fois des cas, l´immuno-hématologie 708 fois, la biochimique 1231 fois et la goutte épaisse 780 fois. Au terme de ce bilan, les patients neurolésés avaient représenté 557 cas dont 345 patients porteurs de lésions cérébrales traumatiques et 212 cas d´accidents vasculaires cérébrales. On notait 71 cas trauma thoraco-abdominal et des lésions plus complexes chez 54 polytraumatisés. Les autres pathologies traumatiques concernaient les brûlures graves 37 cas, les envenimations par morsures de serpent dans 48 cas, les trauma du massif facial 33 cas, les trauma des membres 94 cas.

**Figure 5:**
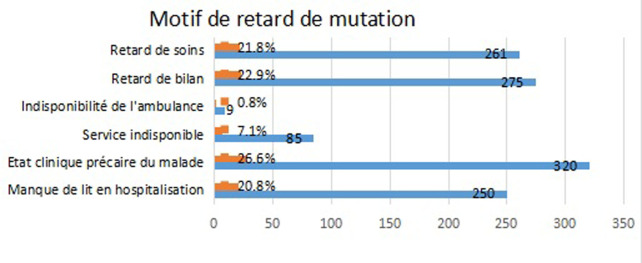
motif de retard de mutation

**Figure 6:**
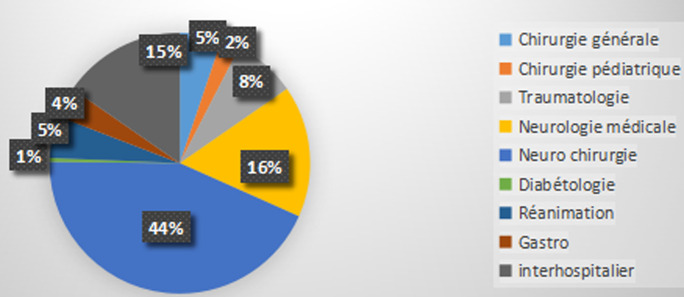
service d´accueil d´aval

Les pathologies médicales étaient dominées par le paludisme grave dans 119 cas, les pathologies infectieuses dont état sepsis dans 81 cas. Les pathologies cardiovasculaires et broncho-pulmonaires ont représenté respectivement 64 et 34 cas. Les complications métaboliques du diabète étaient notées chez 36 cas. Quant aux intoxications médicamenteuses, elles ont représenté 25 cas. Cent soixante-quinze (175) patients étaient des malades médicaux polypathologiques. Le principal motif d´orientation des patients en unité d´hospitalisation de court séjour (UHCD) était l´attente d´un lit d´hospitalisation d´aval dans 89% (n=144/162) des cas. La durée moyenne de séjour dans le service était 63,59 heures (2,65 jours) et une durée max de 45 jours. Quel que soit le motif d´admission, 79,25% (n = 951) des patients séjourneront moins de 03 jours aux urgences, 7% (n=85) y resteront plus de 7 jours. Dans ce dernier cas le profil des patients était dominé par les pathologies médicales. Plus d´un quart des motifs de retard de mutation (Figure 5) intra hospitalière était dû à la nécessite une surveillance spécifique ou des soins particuliers non faisable en hospitalisation conventionnelle. L´écrasante majorité des patients (80%) ont été mutée dans un service à spécialité chirurgicale (Figure 6). Pendant notre période la mortalité globale aux service d´accueil des urgences était de 1, 85%.

## Discussion

La gestion des flux internes a été fait sur la base du triage. Ce processus a servi de base à la sectorisation des patients à l´accueil. Quatre-vingt pour-cent (80%) des admissions ont été orientées dans les box de consultations, conformément aux recommandations de sectorisation des patients [7]. Si cette répartition des patients dans les aires d´accueil était correcte, telle n´avait pas été le cas du circuit du patient dont les causes de dysfonction étaient liées à l´insuffisance de ressource, au délai d´attente d´hospitalisation intra-hospitalier et le refus des services d´accueillir les patients bien qu´une place soit disponible. Pourtant le scandale c´est tous ces patients qui passaient en moyenne 63 heures sur un brancard aux urgences dans des conditions inacceptables. Selon Bruno Riou [7], l´énergie que dépensent les équipes des urgences pour trouver des lits, véritable travail de Sisyphe, peu valorisant et épuisant, détourne une partie non négligeable du temps médical et paramédical qui pourrait être consacré plus utilement à la prise en charge. Si la mission des urgences est de garantir l´accès aux soins d´urgence à tous, il faudra éviter de dépasser le seuil de remplissage critique des structures au-delà duquel il n´y a plus de marges de manœuvre et de capacité d´extension.

le profil des pathologies enregistrés dans notre travail était dominé par les patients neurolésés, il s´agissait de pathologie dont les soins sont lourds et chronophages. Ce type de pathologie était un des facteurs d´engorgement du service en raison de la nécessite un grand nombre d´investigations complémentaires (tomodensitométrie cérébral et une batterie de bilan biologique) dans les suites d´un accident vasculaire cérébral (AVC) ou d´un trauma crânien grave (TCG), du délai d´attente des résultats des examens suscités, des avis spécialisés (elle-même une source de retard de soins). A ces deux sources d´engorgement et de frein à la gestion d´aval des patients venaient s´ajouter éternel manque de lit d´hospitalisation en aval. Un certain nombre de causes de surcharge des services urgences sont bien documentés dans la littérature [8] (consultation non-urgente, les habituées des urgences), ces causes n´avaient pas été retrouvées dans notre travail. La surcharge des services d´urgence est un facteur prédictif indépendant d´augmentation de la morbidité [9,10]. Lorsque l´hôpital est lui-même saturé, la mortalité augmente également, indépendamment de l´âge et de la gravité des patients [11,12]. Les malades des urgences qui étaient en attente d´hospitalisation sont généralement des sujets âgés et polypathologiques; « ceux dont personne ne veut». Dans ce contexte de pénurie de lit d´aval et de précarité clinique, une meilleure alternative était d´encourager le retour au domicile des patients, elle a pu être appliqué chez 30% de nos malades qui sortaient avec une demande de consultation spécialisée. Toutefois l´aberration c´est quand un collègue médecin refuse d´accueillir un patient dans son service sous prétexte que ce dernier est porteur d´une sonde nasogastrique alors même que les familles acceptaient retourner à domicile avec le même patient. Le profil des pathologies allait influencer les services sollicités dans la gestion de l´aval, ainsi le service de neurochirurgie, de neurologie médicale de traumatologie, de chirurgie générale et la réanimation polyvalente ont accueilli 80% des nos patients mutés en intra-hospitalier. Face au surpeuplement, à l´engorgement et la surcharge des services d´urgence plusieurs questions méritent d´être se poser. Faut-il réduire les flux des patients? Doit-on augmenter la capacité d´accueil des urgences ou le nombre de lit d´hospitalisation?

## Conclusion

L´accès à des soins médicaux de qualité en urgence au Mali reste difficile, en raison de l´engorgement des services des urgences. La gestion des afflux massif mérite d’être anticipée. L´organisation de l´orientation des patients en aval des structures des urgences reste le facteur clé pour garantir l´accès aux soins d´urgence. **Implications pratiques**: afin de garantir un accès aux soins d´urgence de qualité face à la demande exponentielle. Promouvoir la régulation médicale pour faciliter et sécuriser l´accès aux soins d´urgence dans les cas requis. Favoriser la coopération entre les structures d´urgence au sein d’un même district sanitaire et inciter le maillage entre les différents niveaux de la pyramide sanitaire. Optimiser le temps de prise en charge des malades en améliorant l´organisation du parcours du patient dans la structure des urgences tout en améliorant les délais de réalisation des examens complémentaires.

### Etat des connaissances sur le sujet

- Faible accessibilité et mauvaise qualité des soins qui sont délivrés aux urgences;
- Augmentation exponentielle de la demande de soins non programmés;
- Engorgement de la structure des urgences.

### Contribution de notre étude à la connaissance

- Au Mali, la gestion des afflux massifs de victime aux urgences est routinière;
- La surpopulation du service des urgences contraste avec le faible taux d´occupation des lits des services d´aval dans le même l´hôpital;
- L´engorgement permanent des services des urgences a favorisé les sortis contre avis médical.
